# Does green tea affect postprandial glucose, insulin and satiety in healthy subjects: a randomized controlled trial

**DOI:** 10.1186/1475-2891-9-63

**Published:** 2010-11-30

**Authors:** Julija Josic, Anna Tholén Olsson, Jennie Wickeberg, Sandra Lindstedt, Joanna Hlebowicz

**Affiliations:** 1 Lund University, Skåne University Hospital, Lund University, Malmö, Sweden; 2 Center for Emergency (JW, JH), Lund University, Skåne University Hospital, Lund University, Malmö, Sweden; 3 Department of Cardiothoracic Surgery, Lund University, Skåne University Hospital, Lund University, Lund, Sweden

## Abstract

**Background:**

Results of epidemiological studies have suggested that consumption of green tea could lower the risk of type 2 diabetes. Intervention studies show that green tea may decrease blood glucose levels, and also increase satiety. This study was conducted to examine the postprandial effects of green tea on glucose levels, glycemic index, insulin levels and satiety in healthy individuals after the consumption of a meal including green tea.

**Methods:**

The study was conducted on 14 healthy volunteers, with a crossover design. Participants were randomized to either 300 ml of green tea or water. This was consumed together with a breakfast consisting of white bread and sliced turkey. Blood samples were drawn at 0, 15, 30, 45, 60, 90, and 120 minutes. Participants completed several different satiety score scales at the same times.

**Results:**

Plasma glucose levels were higher 120 min after ingestion of the meal with green tea than after the ingestion of the meal with water. No significant differences were found in serum insulin levels, or the area under the curve for glucose or insulin. Subjects reported significantly higher satiety, having a less strong desire to eat their favorite food and finding it less pleasant to eat another mouthful of the same food after drinking green tea compared to water.

**Conclusions:**

Green tea showed no glucose or insulin-lowering effect. However, increased satiety and fullness were reported by the participants after the consumption of green tea.

**Trial registration number:**

NCT01086189

## Background

Tea is the second most commonly consumed beverage worldwide after water. Green tea is produced from the plant *Camellia sinensis*. The compounds thought to contribute to the health-promoting effects ascribed to green tea are polyphenolic compounds called catechins [1]. There are four major catechins in green tea: epicatechin (EC), epicatechin gallate (ECG), epigallocatechin (EGC) and epigallocatechin gallate (EGCG), of which EGCG is the most abundant. The focus of many previous studies on green tea has been on the anti-oxidative properties of catechins, and their potential role in preventing cancer and cardiovascular disease [2]. Green tea may also have a beneficial effect on glucose tolerance and the risk of developing diabetes. In a large cohort study on green tea, frequent consumption was found to be inversely associated with the risk of type 2 diabetes among Japanese women [3]. A cross-sectional study in Japan revealed an inverse correlation between daily consumption of green tea at a high concentration and fasting glucose levels in male subjects [4]. Intervention studies with green tea extract (GTE) in healthy rodents [5] and humans [6] have demonstrated increased insulin sensitivity after an oral glucose tolerance test (OGTT) based on lower insulin levels and unchanged glucose levels. Furthermore, EGCG was the catechin found to have most insulin-enhancing activity in an animal *in vitro *study [7]. Several randomized controlled studies investigating the effect of green tea on glucose metabolism have been performed in humans, although with varying results. A crossover trial demonstrated that two months' supplementation with GTE significantly lowered HbA1c in individuals with glucose abnormalities [8]. In contrast, no significant effect on HbA1c was seen after a 3-month trial with GTE supplementation in patients with type 2 diabetes [9]. A crossover study performed on healthy human participants showed that green tea lowered glucose levels after OGTT [10]. In several studies, neither GTE nor EGCG was found to have any effect on fasting glucose, insulin sensitivity or glucose levels after OGTT [11-13].

Changes in lifestyle, such as increased energy intake and decreased physical activity, are causing overweight and obesity, leading to an epidemic increase in type 2 diabetes. Low-glycemic index (GI) diets are associated with lower risk of type 2 diabetes and heart disease [14] and can be useful in the management of glucose levels in patients with diabetes [15]. To our best knowledge, this is the first study to examine the effect of green tea on both the glucose metabolism and satiety, after the ingestion of a regular meal. The primary objective of this study was to determine whether ingestion of a regular meal and green tea lower postprandial plasma glucose levels, glycemic index, and insulin levels. The secondary objective, was to establish whether consumption of a regular meal including green tea increase the satiety. This study was therefore conducted to examine the postprandial effects of green tea on glucose levels, glycemic index, insulin levels and satiety in healthy individuals after the consumption of a meal including green tea.

## Methods

Fifteen healthy subjects volunteered to participate in the study. One subject was excluded on the first occasion due to an inability to ingest the food within the required time. Data were thus collected for seven male and seven female participants [(mean ± SD): age 27 ± 3 years (range 22-35 years); body mass index 22.3 ± 3.4 kg/m^2 ^(range 17.0-30.8 kg/m^2^)]. One subject was a smoker, one was a snuff user and one subject used inhalator agents for asthma. All subjects were recruited from the student population in southern Sweden, and provided their written informed consent. The study was approved by the Ethics Committee of Lund University, and performed according to the Helsinki Declaration. The trial is registered in the US National Library of Medicine with the trial registration number NCT01086189. Subjects received a financial reward for their participation.

The meal consisted of 100 g white bread (*Skogaholms Originalrost*, Bageri Skogaholm AB, Eskilstuna, Sweden) containing 50 g carbohydrates, 8 g protein, 3 g fat, and 2.5 g dietary fiber. In order to resemble a normal meal, 25 g smoked turkey was added, containing 4.5 g protein, 0.75 g fat, and 0.25 g carbohydrates (*Cascina serena*, H. Kemper GmbH & Co. KG, Nortrup, Germany). The total amount of carbohydrates in the meal was 50 g, as recommended by Brouns et al., in their description of GI methodology [16]. The same meal was served with either green tea (green tea meal) or hot water (reference meal). The tea, Japanese *Sencha Makato *(AFTEK Te & Kryddor AB, Arbrå, Sweden) was prepared by brewing 9.00 g of loose-leaf green tea in 300 ml water (initial temperature 80-85°C) for 3 min. The serving temperature of the beverages was 60-65°C. The amount of caffeine in the brewed tea was 26.5 mg/100 ml, and the amounts of catechins were: EC 8.5 mg/100 ml, ECG 29.9 mg/100 ml, EGC <1.0 mg/100 ml, and EGCG 10.8 mg/100 ml.

The design of the study was a crossover randomized control trial without blinding. The study was conducted between 25 January and 11 February 2010. The subjects attended the clinical research department (Skåne University Hospital, Malmö, Sweden) on two different occasions following a minimum 10-h overnight fast. Smoking, snuff taking and medication were prohibited in the morning prior to and during the test. After obtaining a fasting blood sample by finger-prick, venous blood was collected from an indwelling plastic catheter for insulin analysis. The subjects were randomly assigned to either the green tea group or the hot water group, at intervals of at least one week. Each meal was to be consumed within 10 min, after which further blood samples (as described above) were taken at 15, 30, 45, 60, 90, and 120 min after the start of the meal. A validated visual analog score (VAS) was used to assess the participants' subjective satiety on both occasions, according to the method of Hauber et al. based on a scoring system from -10 (extreme hunger) to + 10 (extreme satiety) [17]. A more extensive questionnaire was also used for self-reported ratings on different feelings of satiety. The questionnaires were presented in small booklets showing only one question at a time. The questions asked were: "How hungry are you?" (hereafter denoted "hunger"), "How pleasant would you find eating another mouthful of this food?" ("pleasant"), "How strong is your desire to eat your favorite food right now?" ("desire"), "How full do you feel right now?" ("fullness"), "How sick do you feel right now?" ("sickness"), and "How strongly do you feel that you have had enough to consume?" ("enough"). The subjects were asked to rate the different sensations on a 15 cm VAS anchored by the phrases "Not at all" and "Extremely" [18]. Hunger, desire, sickness and fullness were estimated before the meal (0 min) and 15, 30, 45, 60, 90, and 120 min after the start of the meal. Pleasant was estimated 15, and 30, 45, 60, 90, and 120 min after the start of the meal. The subjects were asked how strongly they felt they had had enough to consume at 15, and 30, 45, 60, 90, and 120 min after the start of the meal. At the same time, the acceptability of the meal was rated on a bipolar hedonic scale, where 1 represents "dislike extremely", 5 represents a neutral response ("neither"), and 9 represents "like extremely".

Capillary plasma glucose samples were collected from all subjects (n = 14), although insulin measurements could not be performed on one subject due to problems associated with vein cannulation. Glucose concentrations were measured with the HemoCue Glucose system (HemoCue AB, Ängelholm, Sweden), which converts blood glucose to plasma-equivalent glucose concentrations by multiplying by a constant factor of 1.11 [19]. The precision of the HemoCue Glucose system is better than ± 0.3 SD between 0 and 22.2 mmol/l. All venous blood samples were centrifuged at 3000 × g for 10 min at 4°C. Aliquots of serum were immediately stored at -25°C for later analysis. Insulin concentrations were measured using an immunoassay with an alkaline phosphatase conjugate (Access Ultrasensitive Insulin, Beckman-Coulter AB, Bromma, Sweden). The sensitivity of the insulin immunoassay was 0.03 mUnit/l (mU/l), and the intra-assay coefficient of variation was less than 10% in the interval 0.03 to 300 mU/l.

### Statistical analysis

The incremental area under the curve (AUC) was calculated for glucose, insulin and satiety for each subject and meal (using GraphPad Prism ver 3.0; GraphPad Software, San Diego, CA, USA). All areas below the baseline were excluded from the calculations. The GI was calculated by expressing each participant's glucose incremental AUC following the test meal as a percentage of the same participant's AUC following the reference meal. Descriptive statistics were run on all measures, and the results are given as means ± SEMs. All statistical calculations were performed using SPSS for Windows software (version 14.0, 2005). Differences in blood glucose, insulin levels, GI, and the questions regarding satiety were evaluated with the Wilcoxon's signed rank sum test. Significance was set at *P *≤ 0.05. This study, employing fourteen healthy subjects, had an 80% power to detect a 20% change in GI at a level of *P *< 0.05 [17].

## Results

### Postprandial glucose and insulin response

**Figure 1:**
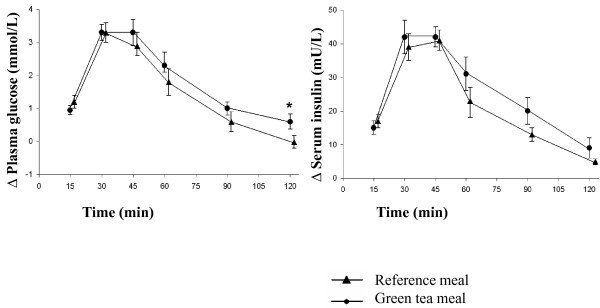
**The mean (± SEM) incremental plasma glucose (n = 14) and serum insulin (n = 13) concentrations in healthy subjects after the ingestion of a green tea meal (•) and a reference meal (▲)**. * Significant difference between the meals according to Wilcoxon's signed rank sum test (*P *≤ 0.05).

**Figure 2:**
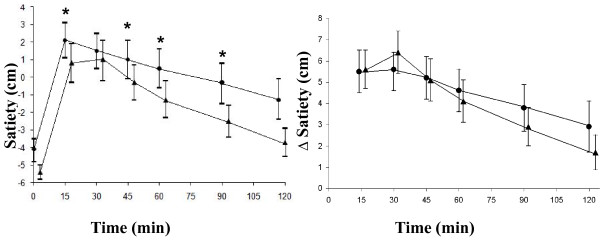
**The mean (± SEM) satiety scores and incremental satiety scores in fourteen healthy subjects after the ingestion of a green tea meal (•) and a reference meal (▲)**. * Significant difference between the meals according to Wilcoxon's signed rank sum test (*P *≤ 0.05).

Ingestion of the green tea meal resulted in a significantly (*P *= 0.019) higher blood glucose response at 120 min than did the reference meal. The postprandial change in glucose level from baseline was also significantly higher after the green tea meal than the reference meal at 120 min (Figure 1). No significant differences were seen in the areas under the plasma glucose curves (Table 1). No significant differences in serum insulin levels or insulin AUCs were observed between the green tea meal and the reference meal during the 120 min postprandial observation period (Figure 2 and Table 1). The mean GI of the green tea meal was 134.2 ± 16.3 (range 66-265) and there was no significant difference compared to the reference meal (GI: 100, *P *= 0.096).

**Table 1:** Postprandial plasma glucose AUC and serum insulin AUC in healthy subjects after the ingestion of a meal with or without (reference) green tea.

	Glucose AUC (mmol·min/l)		Insulin AUC (mU·min/l)	
Time	Reference	Green tea	Reference	Green tea

0-15 min	8.9 ± 1.7	7.1 ± 0.9	111.5 ± 15.6	124.3 ± 18.6

0-30 min	42.9 ± 5.4	39.1 ± 3.3	537.5 ± 60.5	542.8 ± 54.8

0-45 min	89.3 ± 9.3	88.4 ± 7.1	1166.8 ± 109.2	1142.8 ± 88.2

0-60 min	124.2 ± 13.3	127.9 ± 12.8	1711.9 ± 131.6	1619.7 ± 103.9

0-90 min	161.9 ± 21.4	179.5 ± 19.7	2469.5 ± 175.2	2163.9 ± 148.2

0-120 min	177.8 ± 24.7	204.3 ± 22.2	2902.2 ± 224.0	2434.5 ± 168.5

All values are means ± SEM, *n *= 14 for glucose, n = 13 for insulin. No significant differences were found between plasma glucose and serum insulin AUCs following the various meals according to Wilcoxon's signed rank sum test.

### Satiety

**Figure 3:**
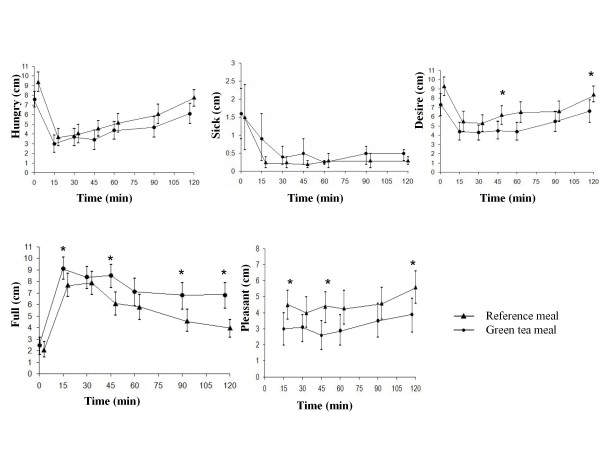
**The mean (± SEM) hunger, sickness, desire, fullness, and pleasantness scores in fourteen healthy subjects after the ingestion of a green tea meal (•) and a reference meal (▲)**. * Significant difference between the meals according to Wilcoxon's signed rank sum test (*P *≤ 0.05).

**Figure 4:**
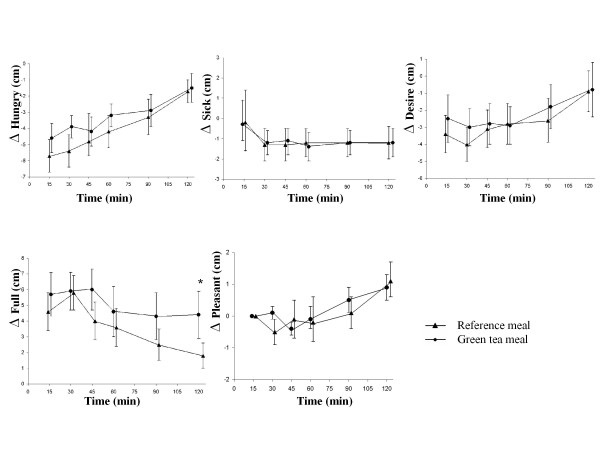
**The mean (± SEM) incremental hunger, sickness, desire, fullness, and pleasantness scores in fourteen healthy subjects after the ingestion of a green tea meal (•) and a reference meal (▲)**. * Significant difference between the meals according to Wilcoxon's signed rank sum test (*P *≤ 0.05).

Ingestion of the green tea meal resulted in a significantly higher postprandial satiety at 15 min (*P *= 0.005), 45 min (*P *= 0.045), 60 min (*P *= 0.025), and 90 min (*P *= 0.030), than the reference meal. However, the postprandial change in satiety score from baseline was no longer significantly different (Figure 2). The AUCs for satiety were not significantly larger at any time after ingestion of the green tea meal than after ingestion of the reference meal (Table 2). Regarding the questions on different aspects of satiety, subjects reported a higher level of fullness after the green tea meal than after the reference meal at 15 min (*P *= 0.054), 45 min (*P *= 0.050), 90 min (*P *= 0.032) and 120 min (*P *= 0.042). The subjects felt more strongly they had had enough to consume after the green tea meal than after the reference meal at 45 min (*P *= 0.005), 90 min (*P *= 0.041) and 120 min (*P *= 0.034) (Figure 3). The postprandial change in fullness from baseline was also significantly higher after the green tea meal than after the reference meal at 120 min (*P *= 0.033) (Figure 4). The change from baseline (at 15 min) in feeling they had had enough to consume was also significantly higher after the green tea meal than after the reference meal at 30 min (*P *= 0.041). The AUCs for fullness, were significantly greater at 90 (*P *= 0.016) and 120 min (*P *= 0.008) after ingestion of the green tea meal than after ingestion of the reference meal (Table 2). No differences were observed in the intensity of hunger at any of the times studied (Figure 3).

**Table 2:** Satiety and fullness AUCs in healthy subjects after the ingestion of a meal with or without (reference) green tea.

	Satiety AUC (cm·min)		Fullness AUC (cm·min)	
Time	Reference	Green tea	Reference	Green tea

0-15 min	42.3 ± 6.8	41.8 ± 7.6	34.6 ± 9.1	46.4 ± 9.1

0-30 min	127.8 ± 22.7	125.6 ± 21.6	109.4 ± 26.2	140.4 ± 24.1

0-45 min	218.5 ± 35.5	206.7 ± 35.0	185.7 ± 40.6	235.7 ± 38.9

0-60 min	288.7 ± 48.6	280.6 ± 48.6	244.8 ± 56.5	298.8 ± 54.3

0-90 min	397.0 ± 73.0	412.0 ± 77.3	342.7 ± 87.4	478.6 ± 90.9*

0-120 min	478.5 ± 91.6	525.0 ± 105.5	415.8 ± 109.4	629.1 ± 126.3 *

All values are means ± SEM, *n *= 14. * Significant differences between the meals according to Wilcoxon's signed rank sum test (*P *≤ 0.05).

No differences were reported concerning how much the subjects liked or disliked the food; they rated the green tea meal and the reference meal as 4.6 ± 0.4 and 4.5 ± 0.4 (out of 9), respectively. No differences were observed regarding the feeling of sickness (Figure 3). The desire to eat one's favorite food was significantly higher after the reference meal than after the green tea meal at 45 min (*P *= 0.044), and 120 min (*P *= 0.025) (Figure 3). After the reference meal, the subjects reported finding it more pleasant to eat another mouthful of the same food at 15 min (*P *= 0.030), 45 min (*P *= 0.019) and 120 min (*P *= 0.015) than after the green tea meal (Figure 3).

## Discussion

The primary endpoint in this study was the effect of green tea on postprandial glucose and insulin levels. Our hypothesis was that green tea could lower postprandial glucose and insulin levels. In animal *in vitro *studies, green tea increased the basal and insulin-stimulated glucose uptake of rat adipocytes [5], suppressed glucose absorption in the rabbit small intestine [20], and ameliorated insulin resistance by increased expression of glucose transporter IV in rat adipocytes [21]. In addition, EGCG exhibited antidiabetic properties by suppressing gluconeogenesis in rat hepatoma cells [22]. We observed no difference in glucose levels and, contrary to what we expected, the 120 min glucose value was higher following the green tea meal. A similar observation has been reported by Park et al. [23], although they also observed significantly lower glucose levels during the first hour of an OGTT trial with green tea in healthy humans. They suggested that the catechins had a hypoglycemic effect in the intestines, but a hyperglycemic effect later when in the circulation. However, the study by Park et al. included few subjects, and it seems that a crossover design was not used. Tsuneki et al. also reported an immediate glucose-lowering effect of green tea powder after OGTT in healthy humans [10]. Although we studied the immediate effects of green tea on glucose metabolism after the ingestion of a white bread meal, in the absence of similar studies for comparison, our findings that green tea does not lower glucose or insulin levels are consistent with previous long-term intervention studies [11-13]. Previous results on the effects of green tea have been ambiguous, and the discrepancy between results from human and animal studies may reflect species-specific differences. Possible reasons for the lack of positive findings *in vivo *might be individual variations in the bioavailability and metabolism of catechins in humans [2].

The secondary endpoint in this study was the effect of green tea on satiety. Our hypothesis was that green tea not only lower postprandial glucose and insulin levels but also increase satiety. Flint et al. [24] concluded that scoring of sensations such as hunger, satiety, fullness, and desire by VAS can be reproduced, and can therefore be used in single-meal studies. In our study, the VAS rating revealed an overall higher sensation of satiety after the green tea meal than after the reference meal. This is supported by the fact that not only was satiety increased, but also the feeling of fullness and the feeling of having had enough to consume. However, no effect was seen on satiety during a 12-week intervention with GTE capsules in obese subjects on a standardized diet [25]. Several factors may have contributed to our positive findings: we used a crossover design, we examined different sensations of satiety at frequent intervals, and we used green tea in its natural form served as a hot beverage. The taste perception of the green tea in this study may have been responsible for the satiety-promoting effect of green tea and so contributed to a stronger satiety sensation after the green tea meal than after the reference meal. Oral exposure to food is related to an increase in satiety, and a decrease in hunger and desire to eat [26]. Measurements of taste perception of the meals in this study would have provided additional information. However, the participants did not dislike the green tea meal more than the reference meal, nor did they feel sicker during the green tea trial, so the higher level of satiety could not be explained by any unpleasantness produced by the green tea meal. The subjects experienced a stronger desire to consume their favorite food or eat another mouthful of the same food after the reference meal.

Since the same kind and amount of food was ingested at both occasions, greater distension of the stomach is not likely to be the mechanism behind these findings. The postprandial glucose concentration is determined by the rates of glucose formation and clearance. Insulin mediates glucose uptake in the tissues after a meal. Gastric emptying rate (GER), together with other factors, regulates the postprandial glucose response, and a reduction in the GER leads to a lower postprandial glucose concentration. Since green tea did not lower postprandial glucose or insulin levels, we can assume that a reduction in the GER is not a likely mechanism behind increased satiety or fullness. Postprandial changes in hormones may be responsible for the satiety-promoting effect of green tea. However, we did not study changes in hormones in this study. The satiety signaling process is very complex, and involves several gastrointestinal peptides and neurotransmitters [27]. Norepinephrine has an important role in satiety signaling in the hypothalamus [28]. Green tea catechins have been shown to inhibit catechol-*o*-methyl-transferase, an enzyme that degrades norepinephrine in the synaptic cleft [29]. This would lead to prolonged action of norepinephrine, and is one possible explanation of the effect of increased satiety with green tea. However, it is uncertain whether polyphenols can cross the blood-brain barrier [30].

Our study has several limitations, and the results should be considered with some caution. Since the study was not blinded, we cannot exclude the possibility that the findings of greater satiety with green tea could be biased. Furthermore, the effect of green tea on satiety was only a secondary endpoint, and the subjects included were healthy and of normal weight. We may have found more significant differences in fullness and satiety if a larger number of participants had been included in the study. To simplify the comparison of the glucose AUC calculations we present our results in terms of GI. We found no difference in GI with green tea, possibly due to large inter- and intrasubject variations in AUCs. The precision could have been improved if the test and reference meals had been repeated. Standardization of the participants' diet 24 hours prior to the trials could have ensured a more similar glucose tolerance on the two trial days.

## Conclusions

Green tea did not lower plasma glucose, GI or insulin levels in this study. Although of modest sample size, the results of this study suggest that green tea may increase satiety and fullness. Clearly, a large clinical trial involving a great number of overweight and obese subjects is needed to further evaluate effects of green tea on satiety.

## Competing interests

The authors declare that they have no competing interests.

## Authors' contributions

The authors' contributions were as follows: JH, SL, ATO, JJ, and JW contributed to the design of the study; JJ and ATO were responsible for recruiting the subjects and carried out the practical aspects of the study. JJ, ATO, and JH performed the statistical calculations; JJ, and ATO created the graphs. JJ and ATO wrote the first draft of the manuscript and JW, SL and JH made critical revisions of the manuscript. JJ and ATO contributed equally. All authors read and approved the final manuscript.
